# Packages of Care for Epilepsy in Low- and Middle-Income Countries

**DOI:** 10.1371/journal.pmed.1000162

**Published:** 2009-10-13

**Authors:** Caroline K. Mbuba, Charles R. Newton

**Affiliations:** 1 The Centre for Geographic Medicine Research (Coast), KEMRI, Kilifi, Kenya; 2 Clinical Research Unit, London School of Hygiene and Tropical Medicine, London, United Kingdom; 3 Neurosciences Unit, Institute of Child Health, University College London, London, United Kingdom; London School of Hygiene and Tropical Medicine, United Kingdom

## Abstract

In the second in a series of six articles on packages of care for mental health disorders in low- and middle-income countries, Caroline Mbuba and Charles Newton discuss treatment for epilepsy.

*This is the second in a series of articles highlighting the delivery of “packages of care” for mental health disorders in low- and middle-income countries. Packages of care are combinations of treatments aimed at improving the recognition and management of conditions to achieve optimal outcomes.*

**Summary Points**

- Epilepsy is the most common chronic neurological disorder, affecting over 65 million people worldwide, of whom 80% are estimated to live in low- or middle-income countries (LMICs).
- Anti-epileptic drugs are very effective in controlling seizures, but most people with epilepsy in LMICs do not receive appropriate treatment.
- This “treatment gap” is influenced by factors such as limited knowledge, poverty, cultural beliefs, stigma, poor health delivery infrastructure, and shortage of trained health care workers.
- Several studies implementing interventions at the community level (for example, training programs for primary health care workers) have successfully improved the identification of people with epilepsy and reduced the treatment gap.
- The sustainability of these interventions needs to be addressed, however, and efforts must be made to ensure a continuous supply of anti-epileptic drugs.

## Introduction

Epilepsy is one of the most common and widespread neurological disorders. Recent estimates suggest that it accounts for 1% of the global burden of disease [1] and affects over 65 million people [2]. In addition, because the relatives and friends of people with epilepsy (PWE) also bear the burden of this condition, more than 500 million people are indirectly affected by epilepsy [3]. Thus, epilepsy imposes a large economic burden on global health care systems and is a major public health problem in low- and middle-income countries (LMICs) [1].

The World Health Organization (WHO) estimates that 80% of PWE live in LMICs. The incidence and prevalence of epilepsy are thought to be higher in LMICs than in high-income countries (HIC)—the median prevalence in LMICs is 9.5/1,000 compared to 8/1,000 in Europe, although the prevalence varies widely among countries [1],[2]. The incidence of epilepsy in LMICs is thought to be up to five times that in HICs, although there are fewer studies on which to base this estimate. Worldwide, mortality among PWE is two to three times higher than in the general population and it is thought to be higher in LMICs than in HICs although data are scarce [4]. The treatment gap, i.e., the difference between the number of people with active epilepsy and the number whose seizures are being appropriately treated, is high in many LMICs [1]. Overall, 56% (range 7%–98%) of PWE in LMICs remain untreated, with 73% remaining untreated in rural regions compared to 46% in urban settings [5].

The International Classification of Disease (ICD) 10 diagnostic criteria for epilepsy are given in Box 1 [6]. The definition of epilepsy has recently been revised by the International League Against Epilepsy (ILAE) [7], but the original classification of seizures (transient occurrences of signs and/or symptoms due to excessive or synchronous discharge of neuronal activity in the brain) as partial (focal), generalized, or unclassified remains useful [8]. Epilepsy has many different etiologies, with head trauma, central nervous system infections, perinatal problems, and cerebrovascular accidents most commonly identified [1], although in most cases a cause is not found. Focal epilepsies represent a greater proportion of epilepsies in LMICs than in HICs and risk factor analysis has identified many causes that are preventable.

**Box 1. International Classification of Diseases 10 Criteria for Epilepsy [6]**

**“G40.0 Localization-related (focal or partial) idiopathic epilepsy and epileptic syndromes with seizures of localized onset**

Benign childhood epilepsy with centrotemporal EEG spikes

Childhood epilepsy with occipital EEG paroxysms

**G40.1 Localization-related (focal or partial) symptomatic epilepsy and epileptic syndromes with simple partial seizures**

Attacks without alteration of consciousness

Simple partial seizures developing into secondarily generalized seizures

**G40.2 Localization-related (focal or partial) symptomatic epilepsy and epileptic syndromes with complex partial seizures**

Attacks with alteration of consciousness, often with automatisms

Complex partial seizures developing into secondarily generalized seizures

**G40.3 Generalized idiopathic epilepsy and epileptic syndromes**

Benign:

 Myoclonic epilepsy in infancy

 Neonatal convulsions (familial)

Childhood absence epilepsy

Epilepsy with grand mal seizures on awakening

Juvenile:

 Absence epilepsy

 Myoclonic epilepsy [impulsive petit mal]

Nonspecific epileptic seizures:

 Atonic

 Clonic

 Myoclonic

 Tonic

 Tonic-clonic

**G40.4 Other generalized epilepsy and epileptic syndromes**

Epilepsy with:

 Myoclonic absences

 Myoclonic-astatic seizures

Infantile spasms

Lennox-Gastaut syndrome

Salaam attacks

Symptomatic early myoclonic encephalopathy

West's syndrome

**G40.5 Special epileptic syndromes**

Epilepsia partialis continua

Epileptic seizures related to: alcohol, drugs, hormonal changes, sleep deprivation or stress

**G40.6 Grand mal seizures, unspecified (with or without petit mal)**

**G40.7 Petit mal, unspecified, without grand mal seizures**

**G40.8 Other epilepsy**

Epilepsies and epileptic syndromes undetermined as to whether they are focal or generalized

**G40.9 Epilepsy, unspecified”**

Although seizures are its most overt manifestations, epilepsy is associated with significant psychological [9] and psychiatric conditions [10], which have social consequences for everyday living [11]. Psychiatric disorders occur in 25%–30% of PWE [10], with depression being the most common, followed by anxiety disorders, psychoses, and personality disorders [12],[13]. Psychiatric comorbidity appears to be particularly common in some LMICs [14]. Stigma poses a major burden to PWE and their families, interfering with the opportunities that PWE have for employment and marriage [15]. Finally, women with epilepsy are particularly vulnerable to sexual exploitation, physical abuse, and extreme poverty [16].

In this article, we focus on the management of epilepsy in LMICs. We review the evidence from LMICs on the efficacy of treatments and delivery of interventions. Because that evidence is often limited, we also refer to systematic reviews, meta-analyses, and key trials from HICs where appropriate. On the basis of this review, we propose a package of care—a combination of interventions aimed at improving the recognition and management of conditions to achieve optimal outcomes—for epilepsy.

## The Evidence on the Treatment of Epilepsy

The evidence for most aspects of the management of epilepsy is poor in both high- and low-income settings (Table 1).

**Table 1:** The evidence in support of epilepsy treatment.

Epilepsy Management	HICs	LMICs
Detection and diagnosis	Screening questionnaires [58],[59]; Use of medical records [60],[61]; Neurological examination [18]; EEG [17]; MRI, CT scan [19]–[21]	Screening questionnaires [29],[62],[63]; EEG [64]; CT scan [65]; MRI scan in those with abnormal EEG [66]
AEDs	Systematic review of initial monotherapy in adults with partial-onset seizures (carbamazepine, phenytoin, and valproic acid), children with partial-onset seizures (oxcarbazepine), and elderly adults with partial-onset seizures (gabapentin and lamotrigine) [23]. Cochrane reviews of efficacy and withdrawal rates associated with phenobarbital, carbamazepine, phenytoin, or sodium valproate use in patients with generalized or partial onset seizures [24]–[27].	RCTs of carbamazepine and phenobarbital in Ecuadorian [28] and Kenyan adults [29] and in Indian [30] and Bangladeshi children [67]. Observational studies of phenobarbital in China [68], and phenobarbital and phenytoin in India [30],[41].
Surgery	Meta-analysis to identify prognostic indicators of seizure remission after surgery [33].	Overview of more than 1,000 operations for epilepsy in India [69].
Ketogenic diet	Systematic review of trials of the ketogenic diet [37]. RCT of the ketogenic diet in children with AED-resistant epilepsy [70].	Ketogenic diet [38]
Psychosocial and psychoeducational interventions	RCT of a 2-d psychoeducational treatment program [71]. RCT of a 6-wk structured psychoeducational group intervention for adolescents with epilepsy and their parents [72]. RCT of a structured nurse-led intervention [73].	Nonrandomized intervention study that provided leaflets about drug information to Taiwanese adults with epilepsy [74].
Cognitive behaviour therapy	—	RCT of acceptance and commitment therapy in South Africa [75].
Relaxation therapy	Two-phase experimental group investigation of a contingent relaxation treatment program [76].	—

Abbreviations: RCT, randomized controlled trial.

### Detection of Epilepsy

Much epilepsy in the world is not identified, particularly in LMICs, and thus PWE may not benefit from treatment. The diagnosis of epilepsy is based on clinical history alone, but since patients may become unconsciousness during seizures, an independent observer is often necessary. In HICs misdiagnosis occurs in 5%–30% of cases [17], and in LMICs this is likely to be higher. In HICs, clinical examination may identify the cause of epilepsy and help with prognosis [18]. For example, electroencephalography (EEG) may detect epileptic discharges, which support the diagnosis, but is not necessary for confirmation. Although this test lacks sensitivity and specificity [17], it is useful for classification of seizures and epilepsy syndromes. Video electroencephalography (telemetry) is particularly useful for management of intractable epilepsy. Various biochemical tests can help in differential diagnosis and can determine an underlying cause of epilepsy. Computerized tomography (CT) scanning is useful for demonstrating gross abnormalities, haemorrhages, and calcification and for detecting parasitic diseases, e.g., neurocysticercosis [19]. Magnetic resonance imaging (MRI) is more sensitive than tomography in identifying structural abnormalities [20]. It is recommended that patients with refractory epilepsy undergo functional neuroimaging. Finally, single-photon emission computer tomography and positron emission tomography may help locate ictal foci, and are particularly useful in identifying candidates for surgery [21]. Unfortunately, these investigations are rarely available in LMICs.

### Anti-epileptic Drug Therapy

Because the cause of epilepsy is often undetermined, and in most cases the epileptogenic focus cannot be removed, anti-epileptic drugs (AEDs) are used to control seizures. In HICs, AEDs may be considered if the person has had seizures within the past 2–5 y (active epilepsy), but in many LMICs AEDs are only often recommended in PWE with a seizure in the past year. AEDs are very effective in controlling seizures: 75% of those treated may become seizure free; 20%–30% of PWE have spontaneous remission of seizures without treatment [22]. Phenobarbital is the least expensive and most widely used AED. It controls a range of seizure types and is on the essential drug list of 95% countries surveyed by the WHO; phenytoin, carbamazepine, and valproate (also first-line AEDs) are on the essential drug list in 86%, 93%, and 87% of countries, respectively [1]. Many other AEDs have been introduced to HICs but are not widely available in LMICs. Each of these AEDs has a profile of adverse events, which may influence their use.

Few randomized trials have compared the efficacy and effectiveness of these first-line AEDs, and most of these trials were conducted in HICs. In a recent review by the International League Against Epilepsy (ILAE), evidence for the efficacy and effectiveness of AEDs as initial monotherapy was found only in adults with partial-onset seizures (carbamazepine, phenytoin, and valproate), children with partial-onset seizures (oxcarbazepine), and elderly adults with partial-onset seizures (gabapentin and lamotrigine) [23]. In Cochrane reviews, no significant differences were documented in efficacy between phenobarbital, carbamazepine, phenytoin, or valproate in patients with generalized or partial-onset seizures [24]–[27]. In LMICs, carbamazepine was not shown to be better than phenobarbital in Ecuadorian [28] or Kenyan adults [29]. In Indian children, there was no difference in efficacy between phenobarbital and phenytoin [30]. However, questions have been raised about the suitability of phenobarbital because of its adverse events [31], and the ILAE has argued that the status of this drug is based on economic factors, rather than on efficacy and suitability [32].

### Surgery

Surgical removal of the epileptic focus is the only cure for epilepsy. Palliative procedures can also be performed. Surgery requires a thorough evaluation to identify the epileptic focus and the relationship of this focus to other functionally important areas of the brain (for example, the speech centre). A recent meta-analysis identified factors associated with seizure remission following surgery (Table 1) [33]. Because surgery requires sophisticated facilities and specialized staff, it is usually only available in HICs. Nevertheless, surgery is increasingly being performed in LMICs [34].

### Vagus Nerve Stimulation

This technique is proposed for the treatment of refractory epilepsy and for the treatment of PWE who are not candidates for surgical treatment. It is effective in treatment of epilepsy patients with partial seizures [35], but is not widely available in LMICs.

### Lifestyle Changes

Although seizures cannot be prevented by lifestyle changes alone, PWE can nevertheless make changes that improve their lives and give them a sense of control. For example, although many PWE do not know the precipitants for their seizures, inadequate sleep, food allergies, alcohol, smoking, and flashing lights may trigger seizures in some patients and can be avoided. Similarly, exercise is important for many aspects of epilepsy [36], particularly to counteract the side effects of some AEDs. However, exercise can trigger seizures in some patients. Finally, dietary changes may prevent the adverse events of some AEDs—phenytoin interferes with vitamin D metabolism, for example. The ketogenic diet (high-fat, no-sugar, low protein diet) is indicated in specific epilepsy syndromes [37], but requires specialized nutritional support that is often not available in LMICs [38]. Other diets are unproven.

### Psychosocial Therapy

Psychological interventions such as psychotherapy, individual, group, or family counseling, progressive relaxation therapy, and cognitive behaviour therapy have all been used in epilepsy (Table 1). However, the trials were small and were mainly conducted in HICs and a systematic review concluded that there was no reliable evidence to support the use of these treatments [39].

## Delivery of Effective Interventions

Delivery of efficacious interventions in LMICs can only be achieved if PWE are correctly identified. Unfortunately, the symptoms of some types of epilepsy (for example, hallucinations) may not be recognized as part of an illness, particularly in LMICs where epilepsy is interpreted within traditional belief systems. Furthermore, in LMICs, trained personnel for the detection and management of epilepsy and facilities for investigations of underlying causes are scarce [1]. However, nurses, community health workers, and key informants such as teachers can improve the identification of PWE [29],[40], and these PWE can then benefit from various interventions (Table 2).

**Table 2:** Delivering epilepsy treatments.

Step	How	By Whom	In What Settings
Increasing demand for the package	Advocacy campaign with the message: “Epilepsy can be controlled” [1],	Patients and support groups; Community health workers; Nurses and physicians; Traditional healers; Public health personnel [29],[41],[45],[46],[77],[78]	Community meetings; Schools; Media, e.g., radio, newspapers; General practice; Homesteads
Increasing access to the package	Making AEDs available in health facilities [29],[41],[51]; Improving awareness about the access to AEDs in health facilities [29],[46],[51]; Organizing satellite clinics [46],[50]; Collaborating with other stakeholders in the health sector [79],[80]	Ministries of Health [1],[31]; NGOs [79],[80]; Private and voluntary agencies	Primary health centres; Private clinics; Hospitals
Improving recognition of the disorder	Community-based and practice-based screening to identify the patients and causes; Clinical history; Neurological examination; Examination of blood AED levels and parasitic infections [1]	Health care workers [29],[41],[46],[51]; Research assistants; Technicians [45]	Community; Primary health centres; Maternal and child health clinics; District General Hospitals; Referral hospitals
Initiation of evidence-based treatments	Supply of first-line AEDs [29],[30],[41],[68]; Training on principles of AED use	Health care workers with license to prescribe AEDs	Primary health centres; Private clinics; Hospitals
Managing serious or complex cases	Referral to other centres with specialist resources [46]	Specialist health care workers	Centres with medical staff with expertise in epilepsy
Achieving optimal outcomes	Increasing AED adherence to reduce seizures, e.g., adherence management until full control achieved for at least 2 y [29],[45]	Health care workers [45]	Primary health centres; Private clinics; Hospitals; Homesteads
Addressing impacts on other health/social outcomes	Improving quality of life by, for example, psychosocial counseling of the family and PWE [48],[81]; Reducing stigma [15],[50]	Health care workers [45],[77]; Traditional healers [55],[56]; Psychiatrists [46]; Psychologists [47]	Primary health centres; Private clinics; Hospitals; Homesteads; Counseling centres

### Interventions to Ensure an Adequate Drug Supply

One of the factors contributing to the treatment gap in epilepsy in LMICs is the lack of a continuous and affordable supply of AEDs [31],[32]. Phenobarbital is most widely used in LMICs. Other available AEDs such as phenytoin, carbamazepine, and valproate are three, 11, and 16 times more expensive, respectively, than phenobarbital [1]; and are often not available and/or routinely prescribed in peripheral health facilities. Because only a 1–3-mo supply of AEDs is dispensed at any one time, PWE have to attend clinics frequently and the cost of these visits may interrupt adherence. Furthermore, the systems for providing a continuous supply of AEDs, particularly to rural clinics, often fail, which discourages attendance. Studies in Kenya and India have shown that programs that ensure a continuous supply of AEDs can improve adherence [29],[41], thereby reducing the treatment gap. Drug banks set up by nongovernmental organizations (NGOs) in some LMICs to supply hospitals when there is a shortage within the government sector can help to ensure adequate drug supplies but are difficult to sustain.

### Interventions to Educate PWE and Caregivers about Epilepsy

Education of PWE and caregivers about epilepsy is important for several reasons. First, PWE with limited knowledge of epilepsy are at increased risk of the complications of seizures, such as fractures, burns, and accidental death [42],[43]. Second, one of the major conflicts in LMICs is the community's beliefs of the cause of epilepsy, and thus its treatment. In many societies, epilepsy is not thought to be caused by diseases of the brain, but is attributed to traditional beliefs such as spirits [44]. Finally, in LMICs, PWE often have cognitive impairment and psychiatric comorbidity, which impairs their understanding of epilepsy and the need to take AEDs daily. Thus, the care of PWE often falls onto family members and friends.

Although psychoeducational interventions tested in randomized controlled trials in HICs significantly improved knowledge and coping with epilepsy and decreased seizure frequency in HICs (Table 1), there have been no similar trials in LMICs, and the educational interventions required are likely to be different in these settings. There have been some programs that increased the knowledge of epilepsy in LMICs as part of a package of interventions but the components of these programs with the most significant effect on AED adherence, seizure outcome, and self-esteem among PWE are difficult to identify [45]–[47].

Patient support groups are found in many countries and play an important role in educating PWE and their caregivers, as well as in advocacy (http://www.ibe-epilepsy.org/links). In addition to education, PWE and their families may also benefit from targeted psychosocial interventions. For example, a study in a London out-patient clinic provided evidence that the provision of skilled counseling to patients with epilepsy in addition to “routine” therapeutic intervention is useful [48]. Counseling helps to deal with many of the problems associated with epilepsy that are not related to the medical or technical aspects of seizure control. Unfortunately, the resources needed for counseling PWE and their caregivers are often not available in LMICs and the provision of such services competes with other health needs.

### Community-Based Interventions to Improve Awareness

Public education is generally advocated as the best method to reduce the stigma attached to conditions such as epilepsy, but interventions need to be based upon qualitative and quantitative assessments to identify the causes of stigma in each region [15]. Information, education, communication, and social marketing are needed to enhance compassion and reduce blame [49]. Community programs to achieve these aims must be based on the local perceptions of epilepsy and needs and must consider social and cultural conditions in the region. To date, community programs have met with some success in Kenya [29], Ecuador [28], Malawi [50], Ethiopia [51], and India [41]. Community-based studies in Kenya and India that supplied free AEDs reported low rates of withdrawal and good adherence and response to therapy [29],[41]. In Ethiopia, community awareness and a regular supply of phenobarbital increased the number of patients attending the clinic [51]. In Malawi, the model tested included publicity of accessible services, adequate supplies of AEDs, setting up of mobile clinics, and frequent follow up by health workers [50]. Unfortunately, most of these programs do not appear to have been sustained since they relied on external funds. Thus, epilepsy care needs to be integrated into primary health care delivery, perhaps with patients providing some of the cost of AEDs to enhance sustainability [45].

The Global Campaign Against Epilepsy, a partnership between the WHO, ILAE, and the International Bureau for Epilepsy was launched in 1997 to improve the acceptability, treatment, services, and prevention of epilepsy worldwide [52]. Several demonstration projects are currently underway under the auspices of this Campaign that aim to reduce the treatment gap and the physical and social burden of epilepsy through community-level interventions. The most successful is the Chinese project, which started with a media-based education program to create awareness about epilepsy and to convey the message that epilepsy is treatable. The program provided free phenobarbital at local health centres and patients were followed up by primary health care physicians who had received basic training in the diagnosis and management of epilepsy [53]. Lectures and group discussions for PWE and their families were arranged and community leaders and teachers were given information about epilepsy. This project improved adherence to AEDs and significantly decreased the treatment gap from 63% to 50% [53].

### Interventions to Train Health Care Providers

There are few specialists, particularly neurologists, in LMICs [1] and health care providers are often ignorant about epilepsies, particularly about their causes, diagnosis, treatment, and psychosocial aspects. Interventions to train health care workers should, therefore, equip them with the knowledge and skills needed to diagnose and manage epilepsy, to counsel PWE, and to make appropriate referrals. It should promote strategies to ensure adherence and follow-up care [46]. Training facilities often cannot be established in LMICs because of the expense [1]. Thus, the training of health care providers in such countries requires the production and distribution of educational materials, including standard guidelines for diagnosis and care of PWE. Our review indicates that educational interventions that targeted health care providers in Zimbabwe and Ethiopia greatly improved the diagnosis and management of epilepsy [45],[51].

### Interventions to Involve Traditional Healers

Despite important advances in the understanding and treatment of epilepsy, many communities in LMICs still believe that epilepsy is supernatural or sacred and is associated with possession, impurity, contagion, heredity, and madness. In many parts of Africa and Asia, notions about epilepsy are rooted not in a medical model but in a spiritual model [54]. Often these beliefs involve external factors and so PWE seek a contextually relevant cure from a traditional healer that removes the alien factor from the body rather than seeking preventative or biomedical treatment.

Traditional healers live within the community and know the communities' perceptions of ill health. Their concentration is much higher in many populations than medical staff [55], and they provide explanations that members of the community believe. They can also provide psychosomatic support, spend longer with their clients than medical staff, and will accept flexible payment systems [56]. But as a group of health care providers, their training, expertise, and beliefs are very variable, and their activities rarely regulated. Nevertheless, because they are frequently consulted in many LMICs, involving traditional healers in the management of epilepsy might be a useful intervention.

### Interventions to Integrate Epilepsy Care into Existing Health Services

Acceptance of epilepsy treatment may be markedly improved by integrating it into existing health care services. Mental health services are particularly important in LMICs since there are more psychiatrists than neurologists and PWE often have considerable psychiatric comorbidity. Experience in both Kenya and Malawi has shown that although epilepsy care can be successfully provided in LMICs, it is much harder to sustain it when it is not integrated into such services [29],[50]. Programs that address epilepsy alone are unlikely to be sustained, and there is a compelling argument that such programs should be incorporated into mental illness or chronic disease services. The sustainability of these programs depends on community participation in planning, implementation, and evaluation as well as integration into primary health care [31]. A study conducted in Ethiopia illustrated how an existing health care infrastructure developed for treatment of infectious diseases could be adapted to deal with epilepsy [51]. An Indian study showed that management of epilepsy should be set within the context of rehabilitation and incorporated into wider programs aimed at the alleviation of poverty [57]. In addition, a commitment needs to be made to deal with the many preventable causes of epilepsy in LMICs such as poor perinatal care, head trauma, and parasitic infections, by integrating epilepsy into wider public health programs [31].

Epilepsy services could also be improved in the community through approaches such as “extension services” (satellite clinic model) of apex institutions and collaboration with other organizations (Table 2). Satellite clinics facilitate the provision of services to the neglected populations of rural areas by reducing the distances travelled by PWE to attend health facilities. Similarly, the creation of partnerships between governmental, private, and voluntary agencies, has proved a useful, cost-effective, and sustainable way to improve the identification and management of PWE [41],[46]. More specifically, in two studies in India where workers in NGOs that were already involved in community-based health care received training in case ascertainment and in informing communities about epilepsy, the epilepsy service continued after the studies ended because it was integrated into existing health care provision [41],[57].

## Packages of Care for Epilepsy in LMICs

Epilepsy, one of the most common neurological conditions, is under-resourced and undertreated in LMICs. A large number of people have significant morbidity and mortality because of the failure to identify cases, difficulties with infrastructure, and the unavailability of suitable AEDs. Education of the community and of health care workers will improve the identification of PWE and thus reduce the treatment gap, provided inexpensive and reliable supplies of AEDs are available. Governments in LMICs need to recognize the burden of epilepsy and need to develop packages of care to reduce the disability associated with this condition in an efficient, sustainable, and equitable manner. We propose two packages of care based on the availability of resources (Table 3). Ideally, the delivery of these packages should be integrated into existing primary health care with the help of NGOs and other nonmedical staff involved in community-based and mental health care.

**Table 3:** Packages of care for epilepsy.

Low Resourced Settings	High Resourced Settings
Epidemiological surveys; Key informants such as community health workers and teachers trained in identification	Primary health care workers, doctors, and neurologists trained in identification and diagnosis
Nurses and clinical officers trained in diagnosis	Specialists in epilepsy
Educational and psychoeducational interventions	Educational and psychoeducational interventions
Limited choice of inexpensive AEDs; Continuous supply of AEDs; Generic formulations	Wide choice of AEDs
Limited services for epilepsy surgery	Services for epilepsy surgery; Ketogenic diet
Health care workers trained in psychological support and counseling	Health care workers trained in psychological support and counseling
Advocacy by NGOs	Advocacy by NGOs

## Abbreviations

AED: anti-epileptic drugs
EEG: electroencephalography
HIC: high-income countries
ICD: International Classification of Disease
ILAE: International League Against Epilepsy
LMIC: low- and middle-income countries
NGO: nongovernmental organization
PWE: people with epilepsy
